# Characterization of membrane protein interactions by peptidisc-mediated mass photometry

**DOI:** 10.1016/j.isci.2024.108785

**Published:** 2024-01-04

**Authors:** John William Young, Emanuel Pfitzner, Raman van Wee, Carla Kirschbaum, Philipp Kukura, Carol V. Robinson

**Affiliations:** 1 Department of Chemistry, Kavli Institute for Nanoscience Discovery, Dorothy Crowfoot Hodgkin Building, University of Oxford, South Parks Road, Oxford OX1 3QU, UK; 2 Department of Physiology, Anatomy and Genetics, University of Oxford, South Parks Road, Oxford OX1 3QX, UK

**Keywords:** Biochemistry methods, Biotechnology, Membranes

## Abstract

Membrane proteins perform numerous critical functions in the cell, making many of them primary drug targets. However, their preference for a lipid environment makes them challenging to study using established solution-based methods. Here, we show that peptidiscs, a recently developed membrane mimetic, provide an ideal platform to study membrane proteins and their interactions with mass photometry (MP) in detergent-free conditions. The mass resolution for membrane protein complexes is similar to that achievable with soluble proteins owing to the low carrier heterogeneity. Using the ABC transporter BtuCD, we show that MP can quantify interactions between peptidisc-reconstituted membrane protein receptors and their soluble protein binding partners. Using the BAM complex, we further show that MP reveals interactions between a membrane protein receptor and a bactericidal antibody. Our results highlight the utility of peptidiscs for membrane protein characterization in detergent-free solution and provide a rapid and powerful platform for quantifying membrane protein interactions.

## Highlights

• Peptidiscs are a powerful platform to study membrane proteins with mass photometry
• Mass photometry (MP) provides a rapid assessment of protein sample homogeneity
• MP can quantify binding between protein ligands and membrane protein receptors
• MP is very useful for revealing stoichiometries for protein-protein interactions

## Graphical abstract

**Figure:**
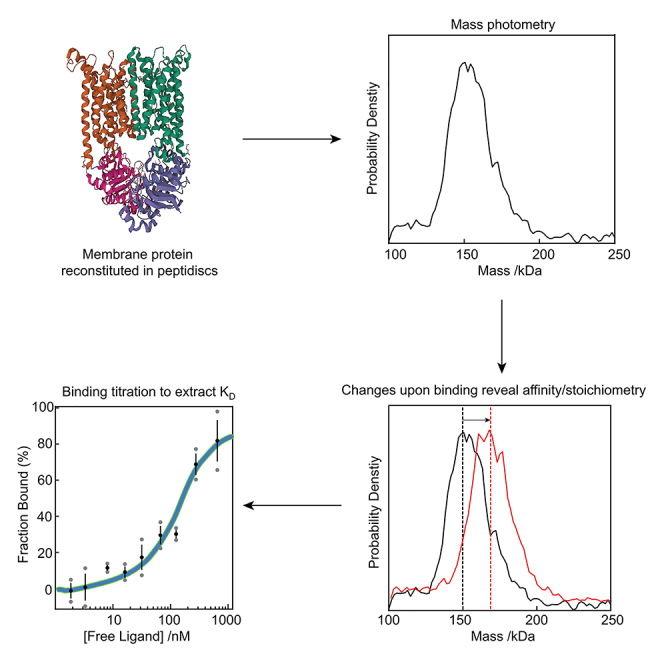

## Introduction

Integral membrane proteins are a highly relevant class of biological molecules and control many of the essential processes of life including energy production, nutrient import, cell-cell signaling, and protein translocation and comprise 60% of current drug targets.^1^^,^^2^^,^^3^^,^^4^ However, membrane proteins are notoriously difficult to study because of their relatively low abundance and high hydrophobicity. Before being analyzed by most structural and/or biochemical techniques, membrane proteins must first be removed from hydrophobic cellular membranes.^5^ Membrane protein extraction from cellular membranes is most often achieved using detergents, which solubilize the lipid bilayer and maintain membrane proteins in a soluble state by shielding their hydrophobic surfaces from water, thereby preventing aggregation. However, even the mildest detergents can disrupt native protein conformations and/or dissociate transiently associated complexes, leading to loss of potentially relevant protein-protein and protein-lipid interactions.^6^^,^^7^^,^^8^^,^^9^^,^^10^

To minimize these detergent effects, numerous membrane mimetic systems have been developed in recent years, which stabilize membrane proteins in a water-soluble format by shielding their hydrophobic transmembrane regions from the aqueous environment. Commonly used membrane mimetics for structural and/or biochemical analysis of membrane proteins include nanodiscs, saposins, peptidiscs, and SMALPs.^1^^,^^11^^,^^12^^,^^13^^,^^14^ With different membrane mimetic systems available, how do researchers identify the optimal conditions for reconstituting their protein of interest in detergent-free conditions? This is often a non-trivial task and can involve applying different reconstitution strategies before identifying optimal conditions for downstream structural or biochemical analysis. Post-reconstitution sample quality is often screened using size-exclusion chromatography (SEC), light scattering coupled to SEC (SEC-MALS), analytical ultracentrifugation (AUC), and negative stain electron microscopy (NS-EM).^15^^,^^16^^,^^17^ Although successful, these techniques are time consuming, have relatively low throughput and, apart from NS-EM, consume substantial amounts of valuable protein sample.^15^ Mass photometry (MP), single molecule mass measurement in solution, has recently addressed many of these limitations for soluble proteins and has emerged as a powerful approach for characterizing protein-protein interactions.^18^^,^^19^^,^^20^ When combined with membrane mimetics including nanodiscs and SMALPs, MP can be very useful to rapidly assess the homogeneity of reconstituted membrane protein samples.^21^^,^^22^ However, the utility of MP for quantifying interactions between reconstituted membrane proteins and their soluble protein binding partners has not yet been explored. In addition, the achievable mass resolution in MP ultimately hinges on the heterogeneity of the chosen carrier, which is substantial for most membrane mimetics.

**Figure 1:**
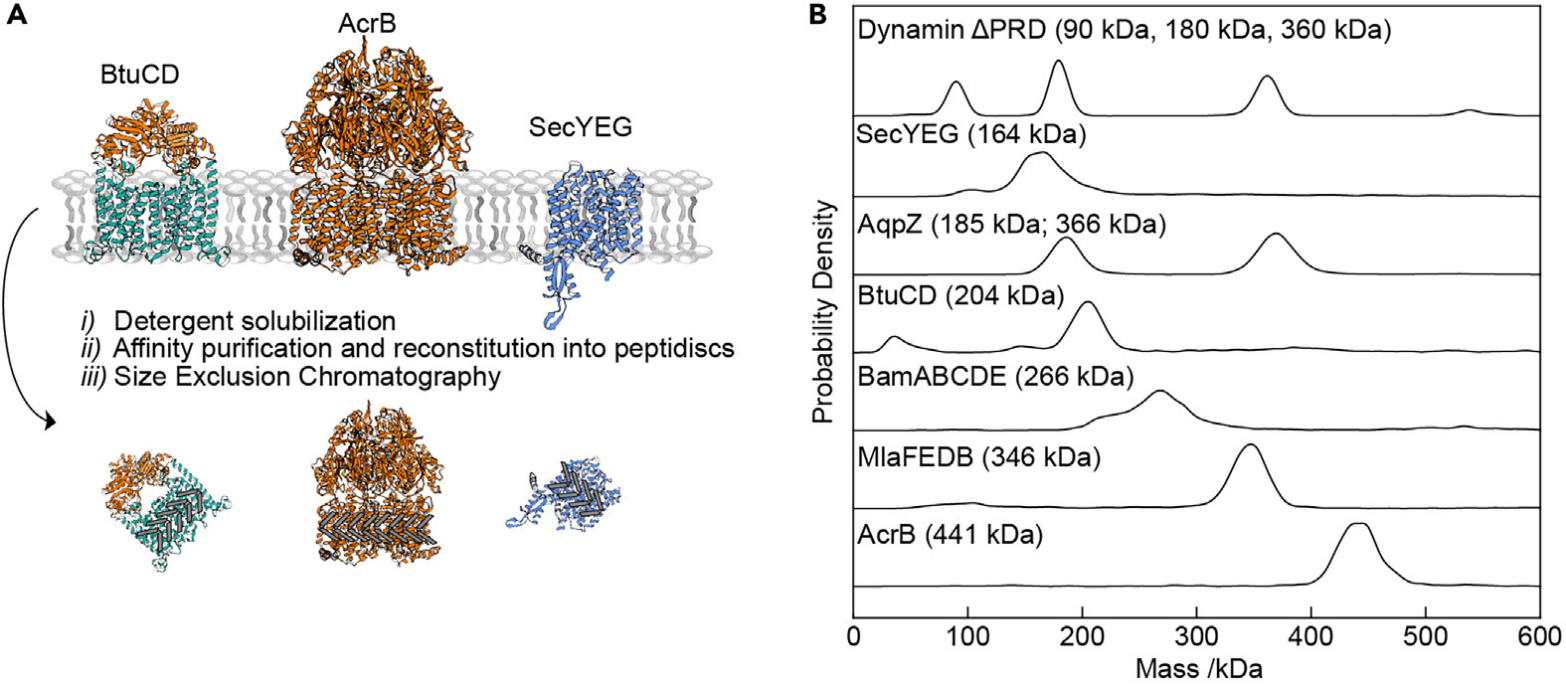
Overview of experimental workflow (A) The overexpressed target protein is extracted from biological membranes with mild detergent (DDM) prior to affinity purification and reconstitution into peptidiscs. Immediately following SEC purification, peptidisc particles are analyzed by mass photometry (MP). (B) Representative mass histograms for each peptidisc-reconstituted integral membrane protein analyzed in this study. The top panel (labeled “Dynamin ΔPRD”) represents the soluble protein standard used to calibrate the MP instrument during these measurements.

Recently, peptidiscs have emerged as an alternative membrane protein carrier featuring a particular straightforward purification procedure.^9^^,^^12^^,^^14^^,^^23^ Here, we use a series of His-tagged bacterial integral membrane protein complexes to demonstrate that membrane protein complexes reconstituted in peptidiscs are amenable to analysis by MP, while exhibiting minimal heterogeneity despite a rapid and simple purification procedure (Figure 1). Using the ABC transporter BtuCD as an example, we demonstrate that MP can be used to quantify high-affinity binding interactions between peptidisc-reconstituted membrane protein receptors and their naturally occurring soluble protein ligands. We further use MP to characterize binding of a bactericidal antibody onto the outer membrane-embedded pentameric BAM complex. These results highlight the utility of the combination of MP with peptidiscs for quantifying protein-protein interactions at the single molecule level.

## Results

### Peptidisc reconstitution of bacterial membrane proteins and analysis by mass photometry

**Figure 2:**
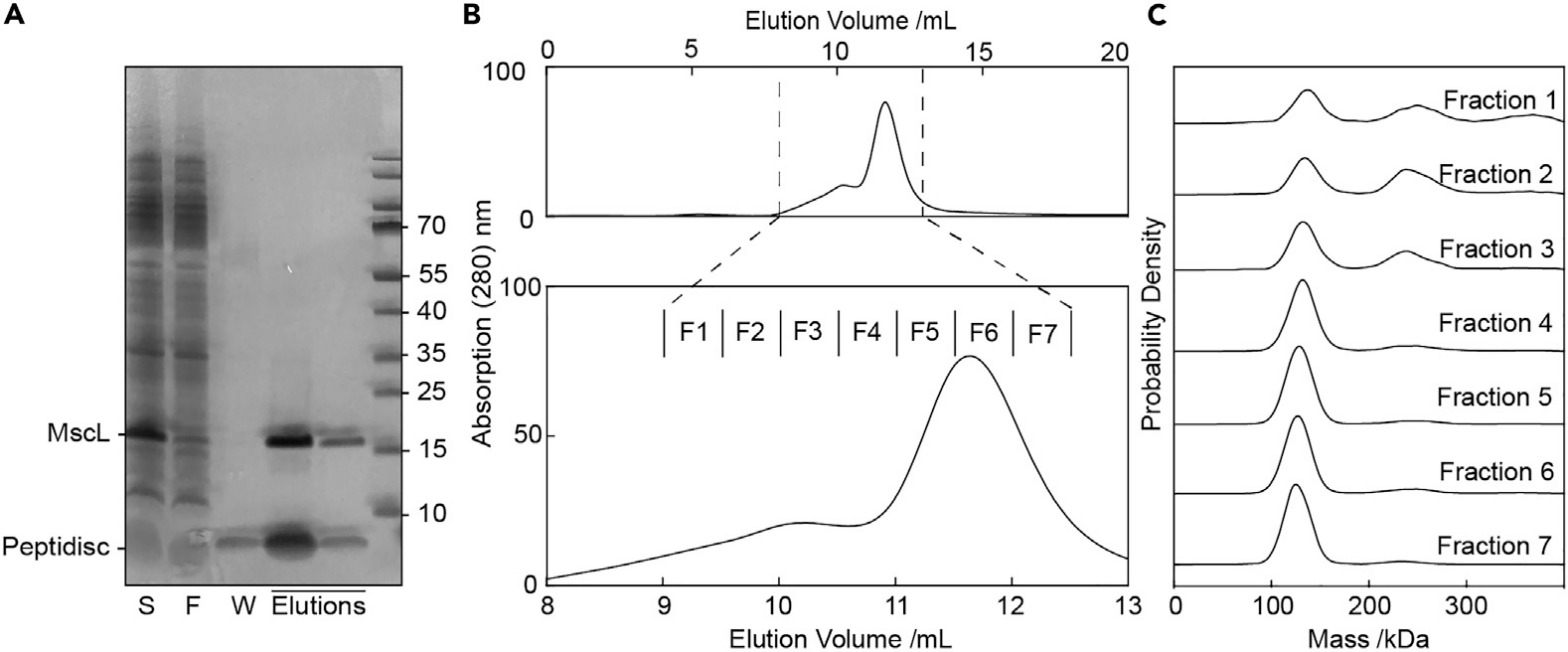
Mass photometry of MscL reconstituted in peptidiscs (A) SDS-PAGE analysis of purified MscL following affinity purification and peptidisc reconstitution. The unpurified detergent-solubilized membrane extract is shown in the leftmost lane (labeled “S”); unbound flowthrough is in lane “F”; peptidisc wash is in lane “W”; purified eluted protein is indicated by “Elutions”; pre-stained molecular weight marker is in the rightmost lane. (B) Size exclusion chromatography (SEC) trace and fractions from (A) on a Superdex 200 10/300 column. Protein absorbance as a function of elution volume was monitored at 280 nm. Multiple fractions (labeled F1-7) were collected and used in further analysis. (C) Each fraction from B analyzed by MP.

To establish our method, we started with the 85 kDa homo-pentameric mechanosensitive channel of large conductance (MscL) from *Mycobacterium tuberculosis* (*M. tb*).^24^ After over-expressing His-tagged MscL in *E. coli* and solubilizing the membrane fraction with DDM, we purified the protein and reconstituted it into peptidiscs. Following SDS-PAGE analysis of the purified material (Figure 2A), fractions were pooled, concentrated, and further purified by size-exclusion chromatography (SEC) in detergent-free buffer. The resulting SEC chromatogram reveals a sharp “major” peak preceded by a smaller “shoulder” peak (Figure 2B). Seven successive fractions under both the “shoulder” and “major” peaks were collected and analyzed by mass photometry (MP). Particle landing events were recorded as movies and analyzed using the DiscoverMP software to produce mass histograms (Figure 2C). The earlier SEC fractions under the “shoulder” peak appear rather heterogeneous, with multiple distinct species present (130 kDa, 250 kDa). The later fractions under the “major” peak, however, appear much more homogeneous, containing a species centered at ∼130 kDa. MscL forms a stable homo-pentamer in cellular membranes, but higher-order clustering has not been observed to date.^24^ Thus, we suspect the higher molecular weight species observed in the earlier fractions may represent non-physiological higher-order oligomers of MscL. Importantly, the resulting MP spectra exhibit highly homogeneous spectra with a full width half maximum (fwhm) < 25 kDa, substantially lower than that achievable with alternative carriers, as reported previously,^21^ which has important implications both for resolution of oligomeric states and quantifying protein-protein interactions.

**Figure 3:**
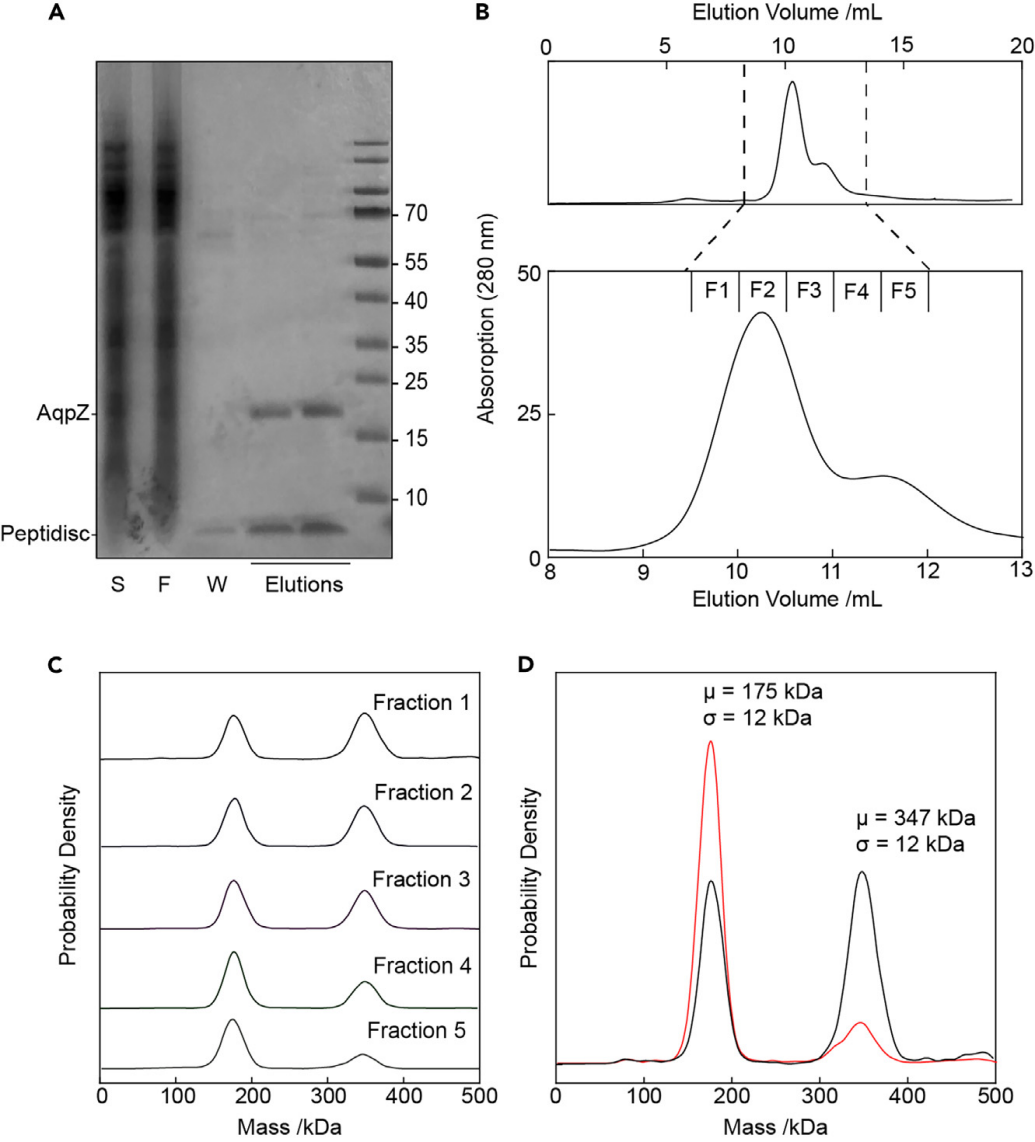
Mass photometry of AqpZ reconstituted in peptidiscs (A) SDS-PAGE analysis of purified AqpZ following affinity purification and peptidisc reconstitution. Gel lanes are labeled as in Figure 2. (B) Size exclusion chromatography (SEC) trace and fractions from (A) on a Superdex 200 10/300 column. Protein absorbance as a function of elution volume was monitored at 280 nm. Multiple fractions (labeled F1–5) were collected and used in further analysis. (C) Each fraction from B analyzed by MP. (D) Fraction 1 from (C) was remeasured in the presence (red trace) or absence (black trace) of 25 mM imidazole. Note that μ = center of mass of each peak; σ = peak standard deviation.

When applying this same workflow to the 100 kDa homo-tetrameric *E. coli* aquaporin AqpZ (Figures 3A–3C), we noticed that the SEC trace was somewhat broader than for MscL. We analyzed 5 successive fractions under the main peak and observed two distinct populations in each fraction: a first sub-population centered at ∼180 kDa and a second centered at ∼360 kDa (Figure 3C). Since the molecular weight of the second species is exactly double that of the first, we rationalized that this may represent two discs stacking together. Indeed, a recent report showed using a combination of SEC and electron microscopy that His-tags can induce formation of non-physiological “stacked” oligomers of membrane proteins.^25^ To test this possibility, we incubated one of the fractions with 25 mM imidazole and repeated the measurement (Figure 3D). In the presence of imidazole, we observed considerably less of the ∼360 kDa species and a correspondingly higher amount of the 180 kDa species, confirming our hypothesis of His-tag induced dimerization occurring with AqpZ.

Previous work from our laboratory and others has shown that both MscL and AqpZ co-purify with annular lipids.^24^^,^^26^^,^^27^ To estimate the phospholipid content of our peptidisc preparations, we performed a phosphorous quantification assay according to the method of Chen et al., 1956,^28^ and found that the total number of phospholipids per disc for the two proteins is similar (Table 1). We further use this information to estimate the total number of peptidisc peptides surrounding each disc, arriving at ∼6 peptides per disc for MscL, and ∼15 peptides per disc for AqpZ (Table 1). We were not surprised to find that the number of peptidisc scaffolds per disc is somewhat different: the AqpZ tetramer consists of 24 transmembrane helices, while the MscL pentamer consists of 10 transmembrane helices.^26^

**Table 1:** Phospholipid content and estimated peptidisc scaffold stoichiometry

Protein Sample	Molecular weight measured by MP (kDa)	Molecular weight of reconstituted protein (kDa)	Ratio of nanomoles lipid: nanomoles disc determined by phosphate analysis	Calculated mass contribution of phospholipid per peptidisc particle (kDa)	Calculated mass contribution of peptidisc scaffold per disc (kDa)	Calculated ratio of peptidisc scaffolds per disc
AqpZ	185	100	24 ± 3	19.2	65.8	15
MscL	130	85	23 ± 1	18.4	26.6	6

The number of phospholipids in each peptidisc preparation (± Std Dev) as determined by phosphate analysis are listed, along with the mass observed using MP and the mass of the reconstituted membrane protein alone. The mass contribution of phospholipids in each sample was calculated by multiplying the number of lipids per disc by 800 Da - the average mass of a phospholipid.^12^

**Figure 4:**
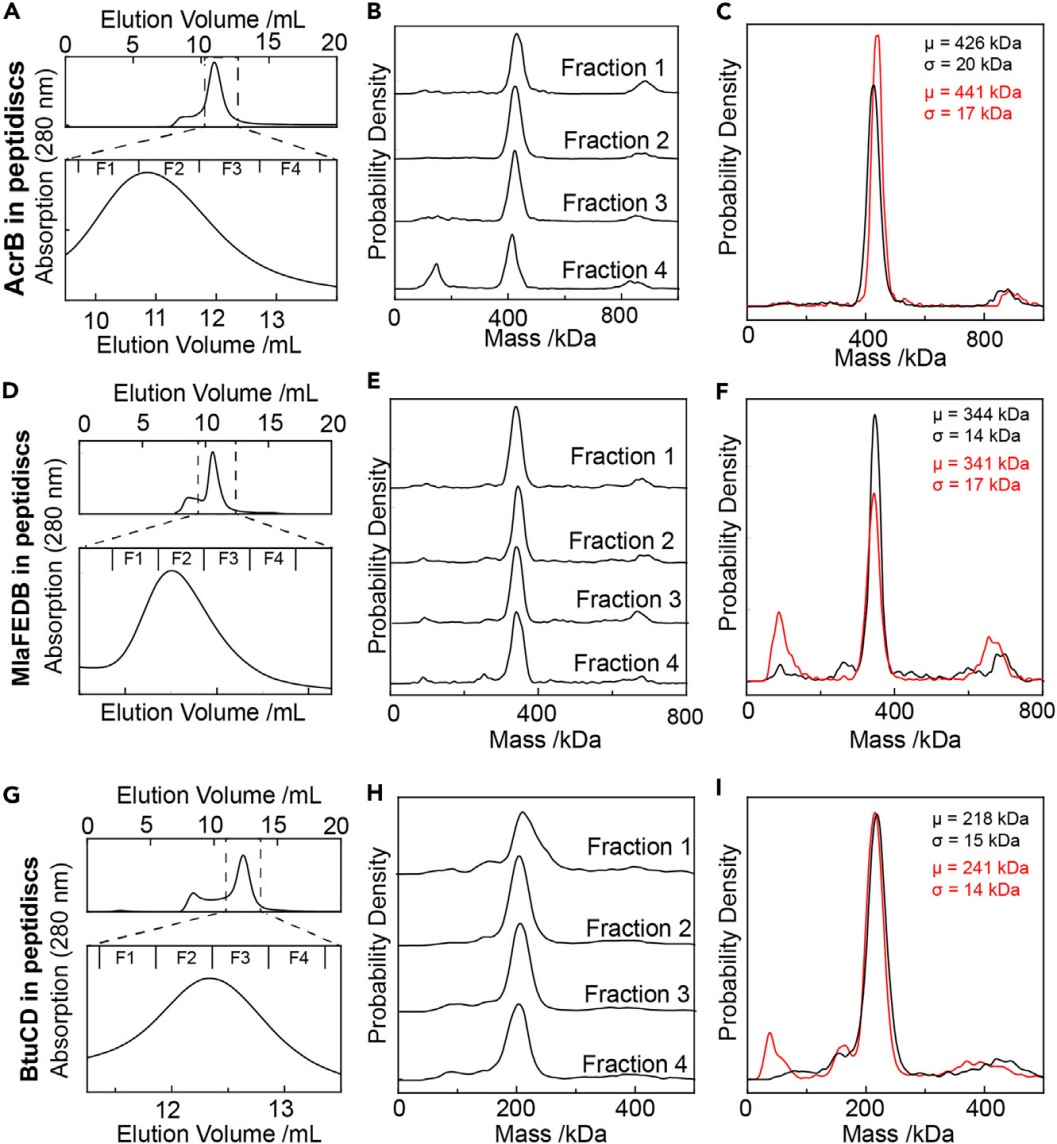
Mass photometry of a selection of integral membrane proteins reconstituted in peptidiscs (A) The trimeric drug efflux pump AcrB was purified and reconstituted into peptidiscs as in Figure 2. (B) MP analysis of the indicated fractions from A, including total number of detected particles per experiment. (C) Reproducibility from two independent biological replicates. (D–F) As in (A–C), but for the ABC transporter MlaFEDB. (G–I) As above, but for the ABC transporter BtuCD. As if Figure 3, μ = center of mass of each peak; σ = peak width.

Next, we applied a similar workflow to the 340 kDa trimeric drug efflux pump AcrB (Figures 4A–4C), the 260 kDa phospholipid ABC transporter MlaFEDB (Figures 4D–4F) and the 130 kDa vitamin B_12_ ABC transporter BtuCD (Figures 4G–4I). Following SEC purification of the reconstituted protein complexes, successive fractions were analyzed by MP. We observed only minimal differences between individual fractions for each complex. For AcrB, each fraction contains a major species centered at ∼440 kDa (Figure 4B); for MlaFEDB, the major species is centered at ∼340 kDa (Figure 4E), and for BtuCD the major species is centered at ∼215 kDa (Figure 4H). The quality of these reconstitutions is highly reproducible, with only minimal differences observed between biological replicates (Figures 4C–4F and 4I).

### Quantifying the interaction of BtuF and BtuCD using mass photometry

We next tested whether we could observe and quantify interactions between peptidisc-reconstituted receptors and their native protein ligands. We started with BtuCD, which interacts with the periplasmic B_12_ binding protein BtuF. Interactions between BtuCD and BtuF have been extensively characterized using detergent micelles and proteoliposomes.^29^^,^^30^^,^^31^ The structure of the BtuCD-F complex has been determined using X-ray crystallography, and the kinetics of the BtuCD-F interaction have been measured using microscale thermophoresis (MST) and surface plasmon resonance (SPR).^29^^,^^30^^,^^31^ These previous studies have shown that BtuF binds BtuCD with high affinity and that the dissociation constant (k_D_) for this interaction is in the nanomolar range.^30^^,^^31^

**Figure 5:**
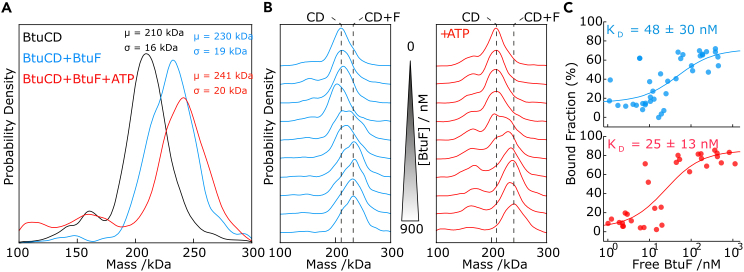
Quantifying interactions between the ABC transporter BtuCD and its protein binder BtuF (A) Representative mass histograms of a fraction under the main SEC peak for BtuCD in the absence (black trace) or presence (blue trace) of a molar excess of BtuF. The “+BtuF” measurement was repeated in the presence of 1 mM ATP (red trace). The masses measured for each sample are indicated in the plot. (B) Incubations of aliquots of BtuCD with increasing amounts of BtuF in the absence (left panel) or presence (right panel) of 1 mM ATP. (C) The fraction of BtuCD that is in complex with BtuF for each condition in B as a function of free BtuF concentration. The points in each plot were fitted to the Hill equation to derive the binding constant (K_D_). Each point represents the mean (±S.D.) for either 6 (-ATP) or 5 (+ATP) independent experiments.

Characterization of BtuCD by MP revealed a species at 210 kDa (Figure 5A, black trace). The 5 kDa difference from our earlier measurement is well within the expected 2% RMS error for independent MP measurements.^19^ The measured mass increases to 230 kDa after incubation with a molar excess of BtuF, indicating that BtuF is binding to peptidisc-reconstituted BtuCD (Figure 5A, blue trace). Given that BtuF is a 30 kDa protein, a partial shift to 230 kDa indicates that not all available BtuCD complexes are bound to BtuF under these conditions. To increase the fraction of BtuCD bound to BtuF, we repeated the measurement in the presence of 1 mM ATP, which promotes BtuCD-BtuF interactions,^31^ increasing the observed mass to 241 kDa (Figure 5A, red trace).

To quantify the binding of BtuF onto BtuCD in the presence and absence of ATP, we titrated BtuF from 0 to 900 nM in the presence of BtuCD (Figure 5B). After an incubation for 30 min, each mixture was analyzed by MP (Materials and Methods). We then plotted the fraction of BtuCD in complex with BtuF by fitting the histograms with two Gaussian distributions (Figure 5C), repeating the titrations multiple times on different days to ensure reproducibility. Combining all measurements and fitting to the Hill equation allowed us to derive a K_D_ for this interaction in the presence and absence of ATP (Figure 5C). In agreement with previous studies, we find that BtuF has a nanomolar affinity for BtuCD, which is modestly increased in the presence of ATP. We further observe that ATP increases the fraction of BtuCD in complex with BtuF.^31^ We remark that the low mass of BtuF enables measurements even at nominally high final ligand concentrations (100 nM) because the increase in background is minimal given that the protein mass is near the detection limit of our instrument.

### Monitoring interactions between the BAM complex and a bactericidal antibody

**Figure 6:**
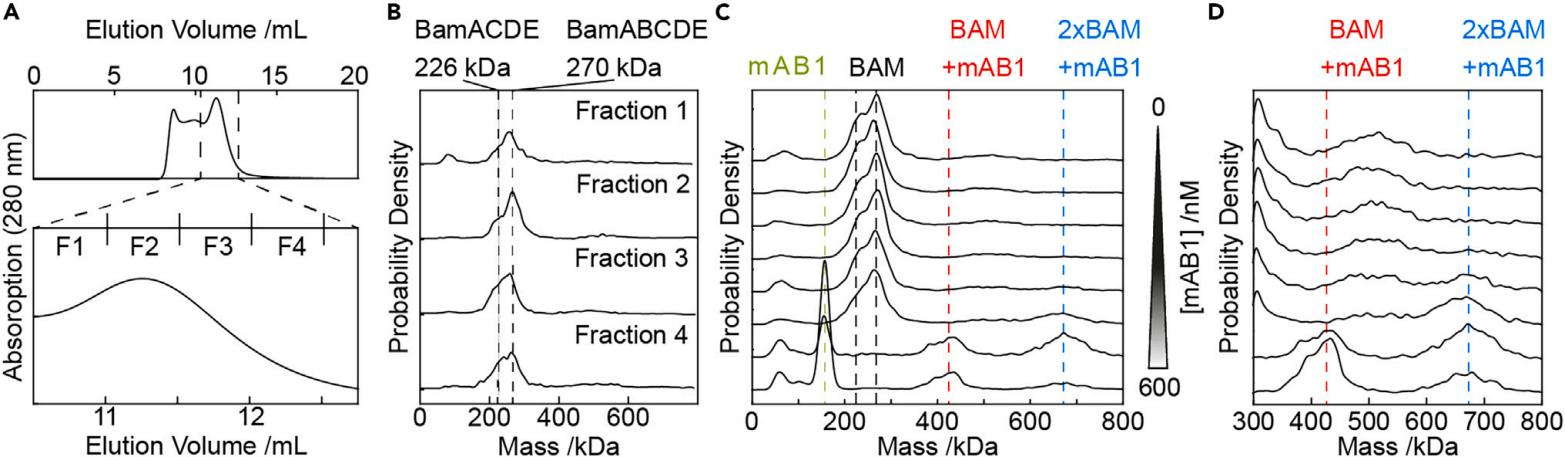
Interaction of the bactericidal antibody mAB1 with the BAM (A and B) Purification and MP analysis of the BAM complex. (C) Incubation of the BAM complex with increasing concentrations of mAB1. mAB1 interacts with BAM to form multiple higher-molecular weight species. (D) The lower MW region (below 300 kDa) was cropped from panel C to highlight changes in the higher MW range (300–800 kDa).

Having verified that our approach is effective for monitoring high-affinity interactions between membrane proteins and their naturally occurring protein ligands, we next assessed whether our method can be extended to monitor interactions between a membrane protein receptor and a therapeutic antibody. As a simple test case, we selected the bactericidal antibody mAB1, which binds to the BamA subunit of the outer membrane-embedded BAM complex.^32^ We purified and reconstituted the 203 kDa BAM complex (comprised of 5 protein subunits, BamA-E) as described previously, and analyzed successive fractions by MP (Figures 6A and 6B). Each fraction appeared to contain two overlapping sub-populations: one at 270 kDa and a second less abundant species at 226 kDa (Figure 6B). The mass difference between the two species is ∼40 kDa, which corresponds to the molecular weight of the BamB subunit. Previous work has shown that BamB is prone to dissociating from the full complex after exposure to detergent.^33^ Thus, the 270 kDa population represents the BamABCDE complex reconstituted in peptidiscs, while the 226 kDa population represents a dissociated sub-complex containing BamACDE. Analysis of the antibody mAB1 alone by MP reveals a single species at 156 kDa (Figure 6C).

We next performed a titration to visualize the binding of mAB1 with BAM. The concentration of BAM was fixed and the concentration of mAB1 increased from 0 to 600 nM and analyzed after 30 min of incubation (Figure 6D). At lower concentrations (0–75 nM) of mAB1, no interaction was evident. At the higher concentrations, however (150–600 nM), we observed two new species at ∼420 and ∼670 kDa. Based on these measured molecular weights, we conclude that the 420 kDa species represents mAB1 binding to one BAM complex, and the 670 kDa species represents mAB1 binding to two BAM complexes (Figure 6D).

## Discussion

In this study, we used mass photometry (MP) to characterize bacterial integral membrane proteins reconstituted into peptidiscs. A feature of peptidiscs that we found particularly advantageous is that membrane protein reconstitution can be seamlessly integrated into a standard affinity purification workflow and requires only minimal optimization to form water-soluble, homogeneous particles (Figures 2 and 4).^9^^,^^12^^,^^14^ When applied to the tetrameric aquaporin AqpZ and the outer membrane-embedded BAM complex (Figures 3 and 6), our method reveals unexpected sample heterogeneity which was not evident during purification. While it is in principle possible to detect and characterize this heterogeneity using SEC-MALS and negative stain electron microscopy, these techniques are laborious, time consuming, and require far more protein sample compared to MP.^21^^,^^25^ Additionally, the resolution obtained here using MP appears much greater than that achievable using either SEC-MALS or electron microscopy.

We previously described the use of MP to characterize membrane proteins reconstituted in other membrane mimetics, including nanodiscs and SMALPs.^21^ Encouragingly, the MP histograms in this report reveals our current reconstitutions are far more homogeneous compared with those reported in our earlier work.^21^ The relatively high homogeneity of our peptidisc preparations was critical for our ability to use MP to monitor receptor-ligand interactions—particularly for small (20–30 kDa) protein ligands.

Using the ABC transporter BtuCD, we show that MP can be used to quantify high-affinity interactions between a membrane protein receptor and its soluble protein ligand (Figure 5). We also used MP to observe binding between the outer membrane-embedded BAM complex and a monoclonal antibody, mAB1, which is bactericidal to *E. coli* under laboratory conditions.^32^ We incubated peptidisc-reconstituted BAM complex with increasing amounts of mAB1 and analyzed the mixtures by MP (Figures 6C and 6D). At high concentrations of mAB1, we observed formation of two distinct higher-molecular weight species—likely corresponding to mAB1 binding to BAM in a 1:1 and 1:2 ratio. While further experiments will be needed to quantify the kinetics of these two distinct binding events, our results show that MP is a powerful method for characterizing binding of therapeutic antibodies onto membrane protein targets.

The combination of peptidiscs and MP presented here has several major advantages compared with other techniques for quantifying receptor-ligand interactions such as biolayer interferometry (BLI), surface plasmon resonance (SPR), and microscale thermophoresis (MST). Our methodology does not require immobilization of one binding partner onto a chip or surface, as is required in BLI or SPR,^22^^,^^23^ nor is any fluorescent labeling required, which is often needed in MST.^31^ In addition, our method unambiguously reveals the stoichiometry of the observed binding events, which can be very useful for characterizing interactions where previous structural or biochemical data are not available, while also easily distinguishing between native and non-physiological oligomerization. These advantages—coupled with the ease of use, the small amount of valuable biological sample required, and the fact that small molecules which may influence the binding reaction can easily be titrated—make our approach very useful for *in vitro* screening of antibody-antigen interactions in the rapidly growing field of antibody discovery against membrane protein antigens.^32^^,^^34^^,^^35^^,^^36^^,^^37^

### Limitations of the study

It is important to note some limitations of our method for quantifying receptor-ligand interactions. First, our method is only effective when the masses of the receptor alone and the mass of the receptor-ligand complex can be differentiated using MP. Thus, while we can use MP to monitor binding of protein ligands and antibodies onto membrane protein receptors, we are currently unable to directly observe binding of small molecules such as antibiotics. For observing small molecule binding onto reconstituted membrane proteins, other methods with greater resolution—such as native mass spectrometry—may be more suitable.^38^^,^^39^ A second drawback is that the receptor-ligand mixtures must be diluted immediately prior to MP analysis. While in this work, we performed a 10:1 dilution, it is equally possible to minimize the degree of dilution by inverting the amount of buffer and analyte, thereby leading to minimal dilution. In all cases, as for all other methods aimed at quantifying affinities from equilibrium, it is important to ensure that the system is measured at equilibrium. In the dilutive approach presented here, this will be the case for off-rates on the order of minutes or faster. For slower off-rates, one can test equilibration by performing the dilution separately, and quantifying the resulting distributions after a time-delay with MP using minimal dilution as described previously, for example after 60 min or more. For such experiments, however, care must be taken to ensure protein loss to container walls is taken into account, which can be quantified by MP directly. In other words, with the exception of long equilibration times for both complex formation or decay (≫hr), MP will provide accurate affinities, while simultaneously informing on the assembly state of its interaction partners.

## STAR★Methods

### Key resources table

**Table:** 

REAGENT or RESOURCE	SOURCE	IDENTIFIER
**Antibodies**		

anti-BamA antibody mAB1	Genentech	Monoclonal antibody 15c9

**Bacterial and virus strains**		

*E. coli* C43 (DE3) competent cells	Cambridge Bioscience	CMC0019
*E. coli* Stellar Competent Cells	Takara	636763

**Chemicals, peptides, and recombinant proteins**		

Peptidisc peptide	Peptidisc Biotech	Peptidisc
Detergent n-Dodecyl-β-D-Maltopyranoside (DDM)	Anatrace	D310S
Adenosine 5’-triphosphate (ATP)	Sigma Aldrich	A26209
Magnesium chloride	Sigma Aldrich	M8266
Deposited Data		
Raw data files	This work	https://data.mendeley.com/datasets/97scsgzkkt/1

**Software and algorithms**		

AcquireMP	Refeyn Ltd.	AcquireMP
DiscoverMP	Refeyn Ltd.	DiscoverMP
GraphPad Prism version 9	GraphPad	GraphPad

**Other**		

Nickel NTA affinity resin	Amintra	ab270549
PBS buffer	ThermoFisher	14190-094
Glass cover slips	VWR	630-2867
CultureWell gaskets	Merck	GBL103250

### Resource availability

#### Lead contact

Further information and requests for resources and reagents should be directed to and will be fulfilled by the lead contact, Prof. Dame Carol Robinson (carol.robinson@chem.ox.ac.uk)

#### Materials availability

Plasmids generated in this study will be shared upon request to the lead contact.

#### Data and code availability

• Mass Photometry data from this study has been uploaded on Mendeley Data and are publicly available as of December 15^th^, 2023. The DOI is listed in the key resources table.
• This paper does not report original code.
• Any additional information required to reanalyze the data reported in this paper is available from the lead contact upon request.

### Experimental model and study participant details

All proteins described in this study were over-expressed in *E. coli* C43(DE3) cells purchased from Cambridge Bioscience. Detailed protocols for cell growth and protein production are provided below (see "Method Details" section). Experiments reported in this paper were performed exclusively on purified proteins from bacteria - thus, neither sex nor gender influenced our results.

### Method details

#### Plasmid preparation and protein expression

Plasmids for expression of *E. coli* BtuCD, BtuF, AcrB and BamABCDE were obtained from our laboratory collection.^40^^,^^41^ BtuF and AcrB are 6x His-tagged on their C-termini; BtuCD is 10x His-tagged on the BtuC subunit. To facilitate expression of *M. Tb* MscL, our laboratory's existing pET15b-MscL-GFP construct was modified using the Polymerase Incomplete Primer Extension (PIPE) method to delete the GFP sequence and add a C-terminal 6x His-tag onto MscL.^24^^,^^26^ To facilitate expression of *E. coli* AqpZ, our laboratory’s existing pET15b-AqpZ-GFP construct was modified using the Polymerase Incomplete Primer Extension (PIPE) method to delete the GFP sequence and add a C-terminal 6x His-tag onto AqpZ.^26^^,^^42^ The plasmid for expression of E. coli MlaFEDB was a kind gift from Dr. Damien Ekiert (NYU).^43^^,^^44^ All plasmids were amplified by transforming them into *E. coli* Stellar Competent Cells (Takara) and the DNA sequences were verified by Sanger sequencing.

For each protein, the appropriate plasmid was transformed into chemically competent *E. coli* C43(DE3) cells (Cambridge Bioscience). A single freshly transformed colony was inoculated into 40 ml LB media supplemented with 100 mg/mL Ampicillin and grown overnight at 37 °C. The next morning, the preculture was diluted 1/100 into 4 L fresh LB media (plus antibiotic) and grown at 37 °C until the culture reached OD600 nm (OD600) between 0.4 and 0.6. Protein production was induced by addition of either 0.2% Arabinose (for MlaFEDB) or 0.5 mM Isopropyl-b-D-1-thiogalactopyranoside (IPTG – for all other constructs), and cultures were grown a further 3 hours at 37 °C. Cells were collected by centrifugation at 5,000xg for 10 min at 4 °C in a Beckman JLA 8.1000 rotor. Excess media was discarded, and cell pellets were resuspended in 20 mL Buffer A (20 mM Tris HCl pH 8, 150 mM NaCl) and stored at -80°C until use. The antibody mAB1 (anti-BamA monoclonal antibody 15c9) was obtained as a lyophilized powder from Genentech under Material Transfer Agreement OR-216904. The powder was resuspended in PBS buffer to a concentration of 5 mg/mL and stored at -80°C until use.

#### Purification of BtuF

Resuspended cells were homogenized by gentle stirring and supplemented with an EDTA-free protease inhibitor cocktail (Roche). The cell suspension was passed three times through an M-110 PS microfluidizer (Microfluidics) at 15,000 psi. Following cell lysis, insoluble material was pelleted by centrifugation at 20,000xg for 20 min at 4 °C in a JA 25.50 rotor. The supernatant was loaded onto a home-packed 5 mL Ni-NTA column equilibrated in Buffer B (Buffer A supplemented with 25 mM imidazole) and allowed to pass via gravity flow. The column was washed first with 50 mL of Buffer B, then with 25 mL of Buffer C (Buffer A supplemented with 80 mM imidazole). Bound proteins were eluted in 20 mL Buffer A supplemented with 250 mM imidazole. After verifying sample purity by SDS-PAGE, fractions containing the target proteins were pooled, concentrated, and further purified by size exclusion chromatography (SEC) on a Superdex 200 10/300 column equilibrated in Buffer A. Peak fractions were pooled and stored at -80°C until use.

#### Purification of membrane proteins and reconstitution into peptidiscs

Resuspended cells were homogenized by gentle stirring and supplemented with an EDTA-free protease inhibitor cocktail (Roche). The cell suspension was passed three times through an M-110 PS microfluidizer (Microfluidics) at 15,000 psi. Following cell lysis, insoluble material was pelleted by centrifugation at 20,000xg for 20 min at 4 °C in a JA 25.50 rotor. To pellet cellular membranes, the supernatant was ultracentrifuged at 200,000xg for 30 min at 4 °C in a Beckman SW32Ti rotor. Membrane protein purification and reconstitution into peptidiscs was performed as previously described with minor modifications.^9^^,^^23^ Membranes were resuspended in 20 mL Buffer A and solubilized with 1% n-Dodecyl-β-D-Maltopyranoside (DDM) for 30 minutes at 4 °C. Insoluble material and aggregates were removed by centrifugation (20,000xg, 20 minutes). The detergent-solubilized material was then loaded onto a home-packed 5 mL Ni-NTA column equilibrated in Buffer A supplemented with 0.02% DDM and allowed to pass via gravity flow. The column was washed first with 50 mL of Buffer B supplemented with 0.02% DDM, then with 25 mL of Buffer C supplemented with 0.02% DDM. To facilitate reconstitution into peptidiscs, the resin was resuspended in 50 mL of Buffer A supplemented with 1 mg/mL peptidisc peptide. Peptidisc peptides were obtained as a lyophilized powder from Peptidisc Biotech.^12^ After collecting excess peptides, the resin bed was washed again with 50 mL of Buffer A. Reconstituted proteins were then eluted in Buffer A supplemented with 250 mM imidazole. After analysis by SDS-PAGE to verify sample purity, peak fractions were pooled, concentrated, and injected onto a Superdex 200 GL 10/300 column equilibrated in Buffer A. Peak fractions were collected. Prior to MP analysis, protein concentration was determined using UV-vis spectroscopy by monitoring the absorbance at 280 nm. To determine the theoretical extinction coefficients for each membrane protein used in this study, the protein amino acid sequence along with the sequence of the peptidisc peptide scaffold was inputted into the Expasy ProtParam tool. The resulting extinction coefficient values are listed in below table.

**Table:** Molar extinction coefficients for the peptidisc-reconstituted membrane proteins used in this study

Membrane Protein	Extinction Coefficient (M^−1^cm^−1^) in peptidiscs
MscL	16960
AqpZ	48930
AcrB	103710
MlaFEDB	92820
BtuCD	120430
BamABCDE	310100

#### Mass photometry analysis of reconstituted membrane proteins

Mass Photometry measurements were performed on cleaned glass cover slips and recorded on a mass photometer (TwoMP, Refeyn Ltd.) as previously described.^18^^,^^21^ In a typical experiment, we first added 5 μL of clean PBS buffer to a silicone gasket (Grace Bio-Labs reusable CultureWell™ gaskets, 50 wells, 3 mm x1mm, Merck Life Science UK Limited) mounted on the clean coverslip to find the glass/buffer interface. To calibrate the instrument, a 20 μL aliquot (at 10 nM concentration) of the globular protein standard - termed "Dynamin ⊗PRD" - was added to the coverslip and a 60 s movie was immediately recorded in either the regular (10.9 x 4.3 μm^2^) or large (16.9 x 12.9 μm^2^) field of view. Following SEC purification of each peptidisc-reconstituted membrane protein, successive fractions were diluted 100-fold in clean PBS buffer to a concentration of ∼5-20 nM. 20 μL of the dilution were added to a fresh well and a 60 s movie was recorded exactly as described for the "Dynamin ⊗PRD" protein standard. The movies were analysed using DiscoverMP software (version 2.5) to quantify protein binding events. Sample molecular weights were obtained by contrast comparison with known mass calibrants measured both before and after each set of experiments. A representative calibration curve is shown in Figure S1. The resulting events were then further analysed and plotted with home written Python scripts using the libraries NumPy, matplotlib, and SciPy.^45^^,^^46^^,^^47^ All mass spectra are kernel density estimates of the resulting histograms with the kernel being a Gaussian of width σ 5 kDa.

To quantify interactions between BtuCD and BtuF, a series of 10 binding mixtures was prepared in 10 μL volume using PBS plus 5 mM MgCl_2_ as the buffer. The concentration of BtuCD was held constant at ∼200 nM across all mixtures, and the concentration of BtuF was increased from 0 – 1200 nM. Following incubation at room temperature for 30 minutes, each mixture was analysed by MP. Immediately before measuring, 2 μL of each was diluted into 20 μL clean PBS and analysed exactly as described above. To test the effect of ATP on the BtuCD-F interaction, the binding titration was repeated with 1 mM ATP present in all conditions. Measurements were repeated multiple times on different days to ensure reproducibility.

To determine the fractions of BtuCD in complex with BtuF, we fitted the mass histograms with 2 Gaussian distributions, one for BtuCD, and one for BtuCD-BtuF (BtuF alone is below the detection limit). For the peak centres of the Gaussian distributions we have used the experimentally determined mass determined for the BtuCD (208 kDa +/- 1 kDa) and used the sequence mass of BtuF (29.4 kDa) +/- 1 kDa to determine the mass of the complex. We have set the sigma of both Gaussians equal to the sigma of the Gaussian fit to the BtuCD histograms (10 - 15 kDa). The optimal Gaussian fits were found using scipy.optimize.curve_fit (Figure S2).

To determine the concentration of free BtuF, we use the following equation.

$$\left[Free\;BtuF\right]=\left[Total\;BtuF\right]-\left[BtuCD-\;BtuF\right]=\left[BtuF\;titrated\right]-f_{Complex}\left[Total\;BtuCD\right]$$

With $f_{complex}$ being the fraction of BtuCD that exists in complex with BtuF, i.e. the ratio between the two Gaussian fits. The area under the Gaussian fit of BtuCD in the absence of BtuF was divided by the total area of the distribution to quantify the fraction of protein of interest in our sample and used to determine [Total BtuCD]. The ‘baseline identification’ of BtuCD-BtuF in the absence of BtuF (caused by the right shoulder of the free BtuCD) was determined and subtracted from all the determined fractions in the titration. Datapoints with an apparent [Free BtuF] < 1 nM were removed, because the number of events under these peaks is too low for reliable quantification.

We have fitted the fraction of BtuCD in complex with BtuF as a function of the concentration of free BtuF with the Hill equation.

$$Complex\;fraction\;\left(K_{D},A,B,\left[L\right]\right)=A+\frac{\left(B-A\right)}{\left[L\right]K_{D}+\left[L\right]}$$

With concentration free ligand defined as [L]; lower asymptote of the fraction of BtuCD in complex with BtuF defined as A; and upper asymptote of the fraction of BtuCD in complex with BtuF defined as B. To extract the K_D_, we have fitted this equation with scipy.optimize.curve_fit, using boundary conditions: 0% ≤ A , B ≤ 100%.

To monitor interactions between the BAM complex and mAB1, a series of 8 binding mixtures was prepared in 10 μL volume, using PBS as the buffer. The concentration of BAM was fixed at 400 nM, and the concentration of mAB1 was increased from 0-600 nM. Binding mixtures were analysed as described above.

#### Phospholipid quantification

The total amount of phosphate present in our peptidisc preparations was determined by a colorimetric assay according to Chen *et. al.*, 1956.^28^ To prepare a standard curve, the following amounts (in nanomoles) of a sodium dihydrogen phosphate solution (NaH_2_PO_4_, 0.91 mM) were deposited in clean glass vials: 0 nmoles; 4.55 nmoles; 9.1 nmoles; 18.2 nmoles; 27.3 nmoles; 36.4 nmoles; and 45.5 nmoles. We then added 112.5 μL 8.9 N H_2_SO_4_ to each tube, before heating them at 200-215°C in a heat block. All tubes were cooled for 5 minutes before addition of 37.5 μL H_2_O_2_. Samples were then heated at 200°C for a further 30 minutes. After cooling to ambient temperature, 1 mL LC-MS grade water was added to each tube, followed by 125 μL 2.5 % ammonium molybdate (VI) tetrahydrate solution and 125 μL 10% ascorbic acid solution. Samples were vortexed thoroughly and heated again at 100 °C for 7 minutes. After cooling to ambient temperature, a 200 μL aliquot of each sample was transferred to a clean plastic cuvette, and the absorbance was measured at 820 nm in a spectrophotometer. Each sample was measured twice to ensure accuracy, and the average value was taken to derive the standard curve. The absorbance was plotted as a function of phosphate concentration in GraphPad Prism 9 and a standard curve was generated by linear regression (Figure S3).

We performed phosphate analysis on 32 μg AqpZ peptidisc, and 60 μg MscL peptidisc. Three glass vials of each sample were analysed to ensure reproducibility. The amount of phosphate in each sample (which corresponds to the total amount of phospholipid in each sample) was calculated based on the standard curve.

### Quantification and Statistical analysis

To quantify binding between BtuCD and BtuF in the presence or absence of ATP, binding titrations were repeated on different days to ensure reproducibility (shown in Figure 5C). A total of 6 independent titrations were performed in the “-ATP” condition; 5 independent titrations were performed in the “+ATP” condition. Each datapoint represents the mean ± standard deviation.

For the phosphate analysis standard curve, each sample was measured twice. Datapoints shown (in Figure S3) represents the mean ± standard deviation. The amount of phosphate in each membrane protein sample shown was measured either 3 (for MscL) or 2 (for AqpZ) times. Data shown in Table 1 represents the mean ± standard deviation for each set of measurements.
