# Supplementary material for: Characterization of membrane protein interactions by peptidisc-mediated mass photometry
Source: iScience. 2024 Jan 4;27(2):108785. doi: 10.1016/j.isci.2024.108785 (PMC10831248; doi:10.1016/j.isci.2024.108785)
Supplement: Document S1. Figures S1–S3 [file mmc1.pdf]

iScience, Volume *27*

Supplemental information

Characterization of membrane protein interactions by peptidisc-mediated mass photometry

John William Young, Emanuel Pfitzner, Raman van Wee, Carla Kirschbaum, Philipp Kukura, and Carol V. Robinson

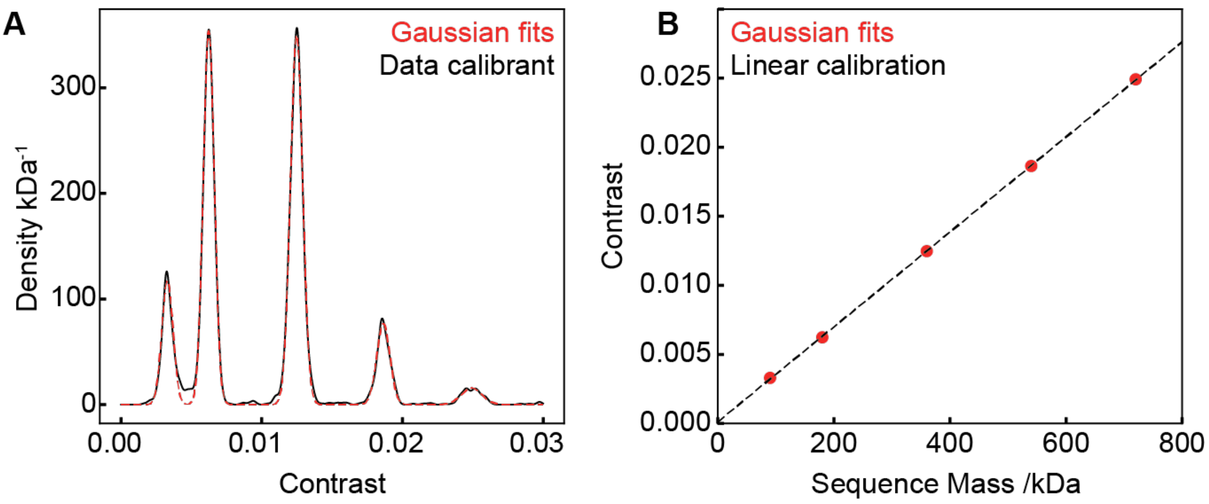

Figure S1 Calibrating the contrast to mass conversion, Related to Figure 1. (A) Contrast distribution (black) and Gaussian fits (red, dashed) for the used calibrant (Dynamin ΔPRD). (B) Contrast versus sequence mass (90, 180, 360, 540 & 720 kDa). The linear fit through the datapoints gives the contrast to mass conversion.

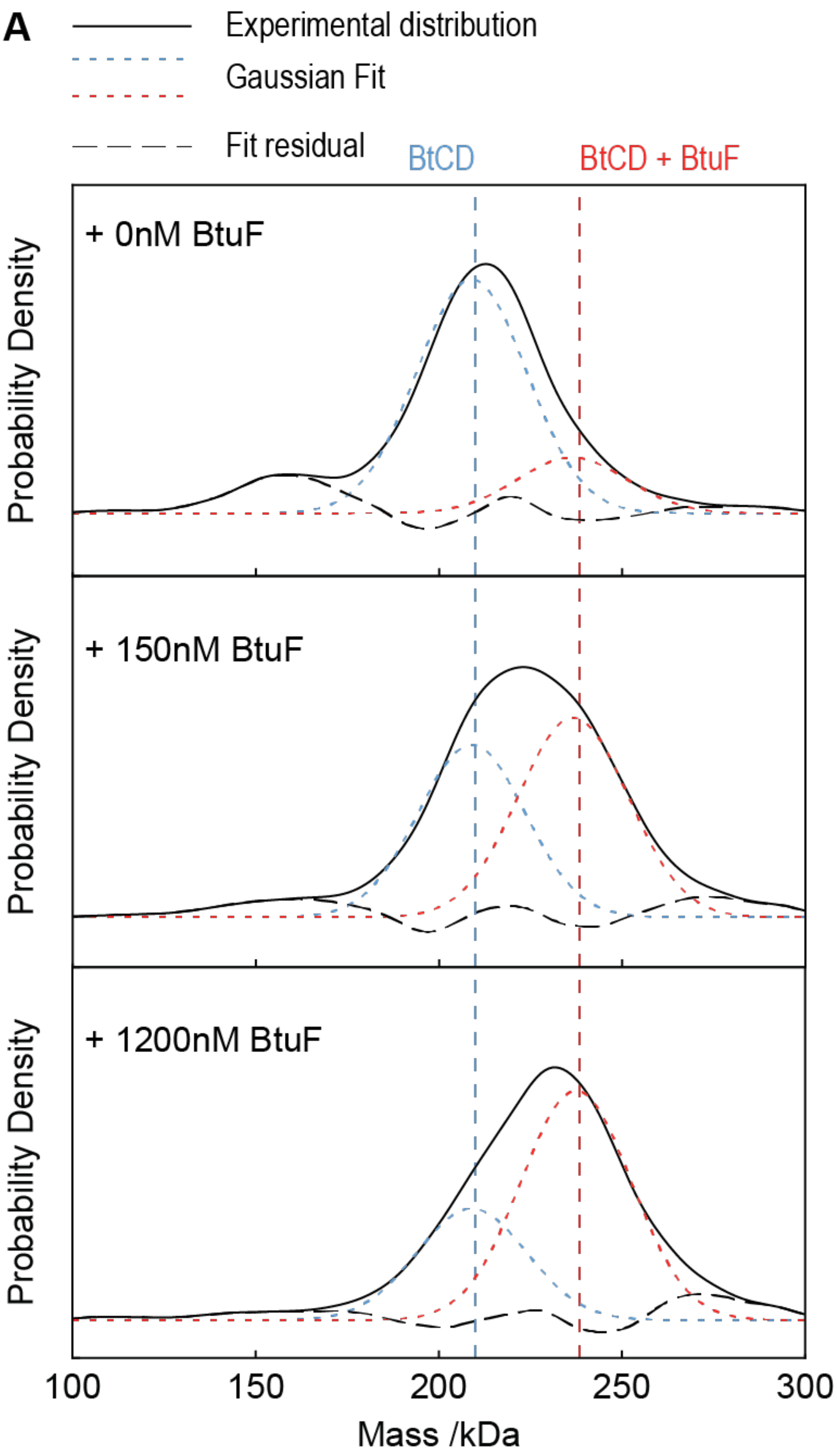

Figure S2 Gaussian fitting to mass distributions in the BtuF & Syd titrations, Related to Figure 5. Experimental distribution (black solid), Gaussian fits (dotted) of the protein (blue) and the protein-ligand complex (red), and the residual of the Gaussian fits (black, dashed) for BtuCD + BtuF for 3 ligand concentrations.

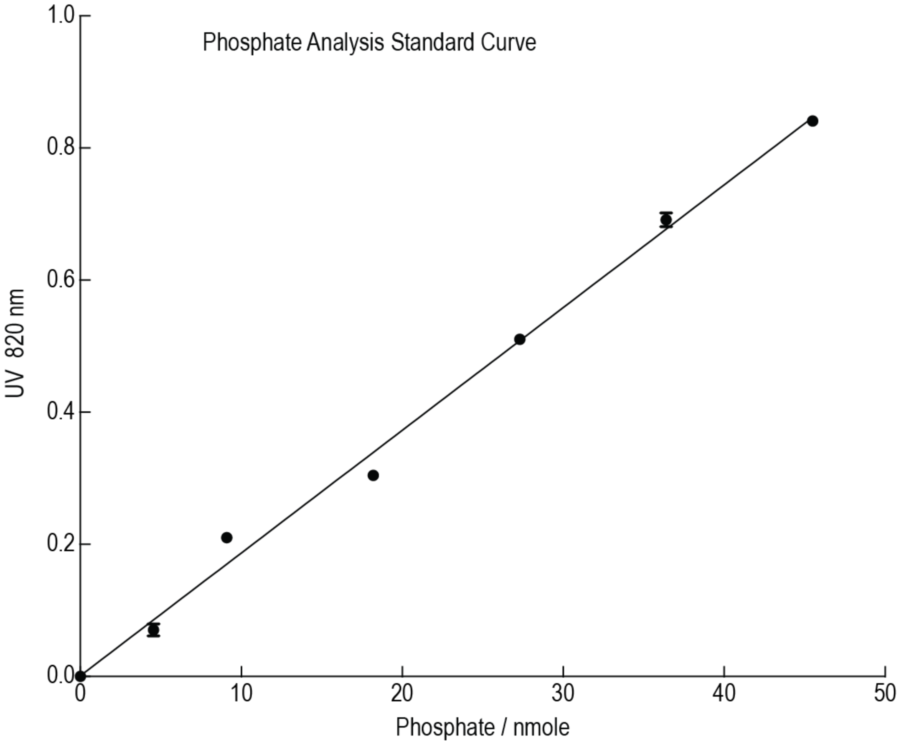

S3 Phosphate analysis standard curve, Related to Table 1. Absorbance at 820 nm plotted as a function of phosphate concentration for a series of 6 solutions of known phosphate concentration. Each point represents the mean of two independent experiments (± S.D.). A standard curve (black line) was generated by linear regression.
